# Transactions of the American Dental Association, 1867

**Published:** 1867-11

**Authors:** 


### TRANSACTIONS OF THE AMERICAN DENTAL ASSOCIATION, 1867.

THIS volume is out *on time*, and makes a fair appearance. It is quite a satisfaction to be able to read it before we have forgotten that there has been a meeting of the Association. It is not a large book; but it contains all the papers referred to the Publication Committee, as well as the official minutes and a synopsis of remarks and discussions. The size of the book, 133 pages, will suggest to the reader who was at the meeting, that the *spoken* proceedings predominated over the *written*. As is usually the case, the *best* of the meeting is the parts that can not be included in the published transactions. We sincerely thank the Committee for the promptness with which the work has appeared, as well as for its creditable appearance. W.

